# Influenza a virus regulates interferon signaling and its associated genes; MxA and STAT3 by cellular miR-141 to ensure viral replication

**DOI:** 10.1186/s12985-023-02146-4

**Published:** 2023-08-18

**Authors:** Mai Alalem, Emad Dabous, Ahmed M. Awad, Nedaa Alalem, Adel A. Guirgis, Samir El-Masry, Hany Khalil

**Affiliations:** Department of Molecular Biology, Genetic Engineering and Biotechnology Research Institute, University of Sadat City, Sadat City, 79 Egypt

**Keywords:** IAV, miR-141, MxA gene, IFN-β signaling, STAT3, IL-6

## Abstract

The antiviral response against influenza A virus (IAV) infection includes the induction of the interferon (IFN) signaling pathway, including activation of the STATs protein family. Subsequently, antiviral myxovirus resistance (MxA) protein and other interferon-stimulated genes control virus replication; however, the molecular interaction of viral-mediated IFN signaling needs more investigation. Host microRNAs (miRNAs) are small non-coding molecules that posttranscriptionally regulate gene expression. Here, we sought to investigate the possible involvement of miR-141 in IAV-mediated IFN signaling. Accordingly, the microarray analysis of A549 cells transfected with precursor miR-141 (pre-miR-141) was used to capture the potentially regulated genes in response to miR-141 overexpression independent of IAV infection. The downregulation of targeted genes by miR-141, in addition to viral gene expression, was investigated by quantitative real-time PCR, western blot analysis, and flow cytometric assay. Our findings showed a significant upregulation of miR-141 in infected A549 cells with different strains of IAV. Notably, IAV replication was firmly interrupted in cells transfected with the miR-141 inhibitor. While its replication significantly increased in cells transfected with pre-miR-141 confirming the crucial role of miRNA-141 in supporting virus replication. Interestingly, the microarray data of miR-141 transduced A549 cells showed many downregulated genes, including MxA, STAT3, IFI27, and LAMP3. The expression profile of MxA and STAT3 was significantly depleted in infected cells transfected with the pre-miR-141, while their expression was restored in infected cells transfected with the miR-141 inhibitor. Unlike interleukin 6 (IL-6), the production of IFN-β markedly decreased in infected cells that transfected with pre-miR-141, while it significantly elevated in infected cells transfected with miR-141 inhibitor. These data provide evidence for the crucial role of miR-141 in regulating the antiviral gene expression induced by IFN and IL-6 signaling during IAV infection to ensure virus replication.

**Supplementary Information:**

The online version contains supplementary material available at 10.1186/s12985-023-02146-4.

## Background

Influenza is a persistent impedance to humanity due to its serious complications during infection, causing seasonal respiratory epidemics every year [1]. Influenza A virus (IAV) belongs to *Orthomyxoviridae* family and is classified into many subtypes according to two main proteins in the virus envelop; hemagglutinin and neuraminidase. Despite the wide spectrum of possible subtypes, only three have continuously existed and releasing pandemics like H1N1 strains in 1918 and 2009 as well as H2N2 in 1957 and H3N2 in 1968 [2].In 1918, the pandemic influenza infection was considered a serious respiratory threat as the pandemic H1N1 virus killed an estimated 50 million people globally and is known as the “Spanish Flu,” but after vaccination, it lost its lethal attitude and became an easy cure infection [3]. Last years, precisely at the end of 2019, the world faced another serious respiratory pathogen, severe acute respiratory syndrome coronavirus 2 (SARS-CoV-2) led to 6.3 million deaths (World Health Organization). Unfortunately, seasonal influenza A viruses (IAV) promote the infectivity of SARS-CoV-2 [4]. Basically IAV genome is composed of negative, eight-segmented; sense single stranded RNA which tightly packed inside the virus core. The three largest segments of the viral genome encode the three viral protein subunits of viral RNA-dependent RNA polymerases (PB1, PB2 and PA) that required for RNA synthesis and replication on host cells. Two RNA segments encode the viral glycoproteins hemagglutinin which mediates binding to sialic acid-containing receptors that facilitate viral entry, and neuraminidase (NA), which is issued for virus progeny release [5]. The RNA segment 5 encodes for viral nucleoprotein (NP) which bound and protect the segmented RNA. RNA segments 6 and 8 encode more than one protein, called, the matrix protein (M1) and membrane protein (M2) and the non-structural protein NS1 (non structure) and nuclear export protein (NEP). Noteworthy, the NS1 protein is a crucial factor that weakens the host antiviral defenses in infected cells. The influenza virus also encodes other helper viral proteins in infected cells, such as PB1–F2 and PA-x, which are required to prevent host innate antiviral responses with the NS1 protein. NS1, NEP, PB1–F2, and PA-x are present in the virus particle but in minimal amounts [6, 7].

Importantly, microRNAs (miRNAs) are small, non-coding RNA molecules that post-transcriptionally regulate gene expression of their target mRNA for finally tuning immune responses [8, 9]. These small sequences (20–25 nucleotides) are transcribed in the form of long hairpin primary RNA (pri-miRNA) by RNA polymerase II [10]. MicroRNAs play a key role on many biological processes, including cell proliferation, cell differentiation, cell death, hematopoiesis, and nervous system patterning [11]. In mammalian cells, miRNAs are up-regulated upon several viral infections to modulate various cellular processes [12]. Cellular miRNAs may enhance viral replication, such as liver-specific miR-122, which was found to be essential for hepatitis C virus (HCV) proliferation. Cellular miR-122 directly interacts with a specific part of the HCV genome, 5´UTR, which contains overlapping *cis*-acting signals involved in translation and RNA synthesis, promoting the expression of viral components [13]. Interestingly, deception of miR-122 using antisense oligonucleotide leads to a dramatic inhibition of HCV reproduction in human liver cell culture, suggesting that antagonism of miR-122 may comprise a novel therapeutic strategy against HCV [14]. Evidence demonstrated that miR-122 expression is significantly reduced in human liver cells treated with IFN-β providing an unknown function of IFN-β to combat HCV infection [15].

Notably, during IAV infection the virus deceives host cellular processes in order to replicate. In one hand Influenza virus inhibits the synthesis of host proteins and facilitates expression of its own proteins and often correlates pathogenicity with growth rate [16]. On the other hand, the infected cells also induced various pathways to inhibit the replication of the influenza virus through a series of complex signal pathways, including PRR-dependent signal pathways and the molecular cascades of RNA helicase retinoic acid-inducible gene I (RIG-I, MDA5, TRAF3, IKK, TBK1), which can induce the production of interferon (IFN) and inflammatory factors, as well as the expression of antiviral IFN-stimulated genes (ISGs) [17, 18]. These proteins not only inhibit the viral replication in infected cells but also stimulate the immune response mediated by T cells and B cells through recruit dendritic cells (DCs) and macrophages from virus-infected tissues [19]. The most prominent antiviral program is controlled by type I interferon, including, IFN-α and IFN-β which restrict multiple levels. Several studies reported that infection with IAV induced the generation of cytoplasmic vacuoles, namely autophagy, at which viral RNA replication complex accumulates and stimulates viral replication [20–22]. The autophagy-related Atg family, such as Atg5, Atg8 (microtubule-associated protein 1 light chain 3, LC3), and Atg12, colocalized with such vacuoles and viral RNA replication complex [23, 24]. Furthermore, the activation of autophagy is initiated by the conjugation between Atg5-Atg12. Such conjugation negatively regulates the IFN-β pathway stimulated upon IAV infection via direct association with RIG-I and IFN-β promoter stimulation 1 (IPS-1) [25].

In this way, we aimed to investigate whether miR-141 is upregulated in human lung epithelial cells (A549 cells) in response to IAV infection and the possible involvement of miR-141 in IAV-regulated cellular immune response stimulated upon infection.

## Methods

### Cell lines

Human lung carcinoma epithelial cells A549 cell lines obtained from (VACSERA, Giza, Egypt) and MDCK (Madin-Darby canine kidney) cell lines were propagated in the Roswell Park Memorial Institute 1640 media (RPMI), containing 25 mM HEPS, 4 mM L-glutamine, and 10% of heat-inactivated bovine serum albumin (BSA) and incubated at 37 °C and relative humidity of 95%. Cell lines were regularly checked for mycoplasma contamination [26].

### Influenza virus strain

To reproduce the influenza virus, IAV A/WSN/33, A/PR/8/34, and A/PDM/08 were injected into the allantoic membrane of embryonated chicken eggs 10-day-old eggs, and then the infected embryos were incubated to allow virus growth. High titers of viruses were then recovered from the infected eggs [27].

### Interferon treatment and infection protocol

A549 cells were seeded in a 6-well plate with a density of 10 × 10^4^ cells/well and incubated overnight at 37 °C and relative humidity of 95%. Before the infection, the old media was discarded, and the cells were washed with phosphate buffer saline (PBS); then, the cells were infected with different MOI (multiplicity of infection) of IAV including, 0.05, 0.1, 0.25, 0.5 and MOI of 1 for one hour at room temperature using PBS supplemented with 0.1% BSA. The infectious media was discarded, and 2 ml of complete RPMI media was added to each well; then the infected cells were incubated for 24 h [20]. To investigate the effectiveness of IFN-β on viral replication, A549 cells were grown in 6-well plates. Cells were then exposed to the IFN-β (5 IU/ml) 2 h before the virus infection and continuously during the infection, whenever needed, noninfected and infected cells were left without IFN-β to serve as controls.

### Transfection protocol

A549 cells were grown in a complete RPMI medium and were overnight cultured in 6-well plates with a confluency of about 80%. A549 cells were then transfected with either a respective inhibitor antagonist miR-141 (5-′ACAACCACTGTCTGGTAAAG-3′) or pre-miR-141 using Lipofectamine LTX (Invitrogen, USA). According to the manufacturer’s instructions, the cells were transfected with 12.5 µg/ml using 20 µl Lipofectamine LTX prepared in 500 µl optimum media. Cells transfected with the same concentration of transfection reagents were severed as control-transfected cells. Transfected cells were incubated for two days and then were infected with IAV (MOI of 1). The knockdown efficiency of miR-141 and the relative expression of indicated genes were monitored in transfected and infected cells using qRT-PCR. Flow cytometry assay was used to detect the kinetic protein expression of MxA and STAT3 in transfected and infected cells [24].

### Proliferation and cytotoxic assay

For proliferation assay of transfected A549 cells with either miR-141 inhibitor or precursor, cells were cultured in duplicate in a 6-well plate at 10 × 10^4^ cells per well. Cell morphology was monitored using the inverted microscope. The number of survived cells upon transfection was accounted by using a hemocytometer. In brief, the old media was discarded, and the cells were washed twice with phosphate buffer saline (PBS) before trypsinizing by 3 min incubation at 37ºC. Then a suitable volume of complete RPMI media was added to the trypsinized cells, and the number of cells was manually accounted [28, 29]. To investigate the cytotoxicity of miR-141 transfection, A549 cells were seeded in triplicate in 96-well plates at 10 × 10^3^ cells per well and were incubated overnight. Cells were transfected with varying concentrations of pre-miR-141 or specific inhibitor (12.5–200 µg/well). Cells treated with transfection reagents served as control-transfected cells. The cell viability rate was monitored by using MTT colorimetric assay kit (Sigma-Aldrich, Germany). In brief, the cultured media was discarded, and PBS was used for washing the cells; then 100 µl RPMI media was added to each well. Next, 10 µl MTT solution was added, and the plate was incubated for 1 h at 37ºC. Finally, 100 µl SDS-HCl was added to each well in the plate which then was incubated for 4 h at 37ºC. Cell viability was monitored depending on the amount of converted water-soluble MTT to an insoluble formazan which is then solubilized and determined by optical density at 570 nm.

### Microarray analysis

For gene expression profiling of transfected cells, total RNA was isolated by the TRIzol Reagent RNA preparation method (Invitrogen) using glycogen as a carrier. Quality control and quantification of total RNA amount were assessed using an Agilent 2100 bioanalyzer (Agilent Technologies) and a NanoDrop 1000 spectrophotometer (Kisker). In brief, mRNA was reverse transcribed and amplified using an oligo-dT-T7-promotor primer, and the resulting cRNA was labeled either with Cyanine 3-CTP or Cyanine 5-CTP. After precipitation, purification, and quantification, 1.25 µg of each labeled cRNA was fragmented and hybridized to human whole genome microarrays, according to the supplier’s protocol (Agilent Technologies). All microarrays were scanned at 5 μm resolution using a DNA microarray laser scanner (DNA Microarray Scanner BA, Agilent Technologies), using Ambion ship and Exiqon ship according to the manufacturer’s instructions. Raw microarray images were analyzed with the Image Analysis / Feature Extraction software G2567AA (Version A.7.5, Agilent Technologies) using almost all default settings for miRNA microarrays loaded with a grid file or standard setting. Non-uniformity outlier flagging was done with a 5x default value of the constant coefficient of variation for features ((CV) 2 Term (A) = 0.55) and background ((CV) 2 Term (A) = 0.45). Intensities were normalized by local background correction and rank consistency filtering with LOWESS normalization. The intensity ratios were calculated by using the most conservative estimate of error between the Universal Error Model and propagated error. Agilent human whole genome arrays were processed using the GE2-v4_95_Feb07 protocol and standard settings according to the supplier’s instructions. The extracted MAGE-ML files were further processed and analyzed with the Rosetta Resolver Bio-software, Build 6.1.0 (Rosetta Bio-software). A color-swap dye reversal was performed to compensate for dye-specific effects and ensure statistically relevant data analysis. Ratio profiles comprising single hybridizations were combined in an error-weighted fashion to create ratio experiments. A 1.5–fold change (whole genome arrays) or 2-fold change (miRNA arrays) expression cut-off for ratio experiments was applied together with anti-correlation of color-swapped ratio profiles, rendering the microarray analysis highly significant (*P* value < 0.01), robust and reproducible.

### Detection of miR-141 relative expression

To detect the expression level of miR-141, small miRNAs was extracted from infected or transfected A549 cells (48 h post-transfection) using PureLink™ miRNA Isolation Kit (Invitrogen, USA) according to the manufacturer’s protocol. RT-PCR was used to detect the relative expression of miR-141 in two steps. First, cDNA was performed from small miRNAs using reverse transcriptase reaction followed by the amplification step using miR-141 and RNU6B specific TaqMan microRNA assays (Applied Biosystem, Darmstadt, Germany), according to the manufacturer’s protocol. Levels of RNU6B were used for normalization. To perform cDNA from small miR-141, the following reagents were prepared for reaction: 0.15 µl dNTPs (100 mM), 1 µl reverse transcriptase (50 U/µl), 1.5 µl 10X reverse transcriptase buffer, 0.2 µl RNase inhibitor (20 U/µl), 5 µl purified miRNAs (10 ng/µl) and 1 µl from miRNA reverse primer up to final volume of 20 µl using RNase free water. The mixture then incubated 30 min at 42 °C followed by 5 min at 85 °C to inactivate the enzyme. The resulting cDNA then was used as a template to amplify both miR-141 and RNU6B by using 0.5 µl from each specific primer presented in Tables 1 and 10 µl SYBR green, 0.2 µl RNase inhibitor (20 U/µl), and 1 µl cDNA (100 ng/µl) to reach a volume of 20 µl using RNase free water. The following parameters were performed in quantitative real-time PCR (qRT-PCR) machine: 95 °C for 5 min, 40 cycles (95 °C for 15 s, 60^°^C for 15 s and 72 °C for 15 s) and 72 °C for 3 min.

**Table 1:** Oligonucleotides sequences used for quantifying miR-141 and mRNA of indicated genes

Description	Primer sequences 5’-3’	
MiR-141-sense		CGCTAACACTGTCTGGTAAAG
MiR-141 antisense		GTGCAGGGTCCGAGGT
MiR-U6-sense		GCTTCGGCAGCACATATACTAA
MiR-U6-antisense		CGCTTCACGAATTTGCGTGTCAT
NP-sense		ATATTGAGAGGGTCGGTTGC
NP-antisense		ATCCACACCAGTTGACTC
NS1-sense		AGTTTCGAACAAATAACATT
NS1-antisense		CCAGATCGTTCGAGTCGT
MxA-sense		GTTTCCGAAGTGGACATCGCA
MxA-antisense		GAAGGGCAACTCCTGACAGT
STAT3-sense		GCCAGAGAGCCAGGAGCA
STAT3-antisense		TGAAGCTGACCCAGGTAGC
GAPDH-sense		TGGCATTGTGGAAGGGCTCA
GAPDH-antisense		TGGATGCAGGGATGATGTTCT

### Detection of relative gene expression

To detect the relative gene expression, qRT-PCR was used to perform cDNA construction and amplification in one step using the purified total RNA as a template. Total RNA from transfected and infected A549 cells were extracted using TRIzol and purified using the RNeasy Mini Kit (Qiagen, USA). The relative expression of viral *NP, NS1, MxA*, and *STAT3* were detected using the QuantiTect SYBR Green PCR Kit (Qiagen, USA) in miR-141 transduced cells or miR-141 depleted cells. The oligonucleotides in Table 1 have been used as specific primers for indicated genes. Level of amplified GAPDH by the oligonucleotides, 5´-ggtatcgtggaaggactcatgac-3´ and 5´-atgccagtgagcttcccgttcag-3´, was used for normalization. The following reagents were prepared for each reaction; 10 µl SYBR green, 0.2 µl RNase inhibitor (20 U/µl), 0.25 µl reverse transcriptase (50 U/µl), 1 µl purified total RNA (100 ng/µl) and 0.5 µl from each primer up to a final volume of 20 µl using RNase free water. According to the manufacturer’s protocol, the following PCR parameters were sued to construct and amplify cDNA, in one step, from a total RNA template: 50 °C for 30 min, 95 °C for 3 min, 40 cycles (95 °C for 30 s, 60 °C for 15 s, 72 °C for 30 s) and 72 °C for 10 min [30, 31].

### Flow cytometry analysis

Flow cytometry was used to assess the kinetic protein expression of NP, NS1, MxA, and STAT3 in transfected and infected A549 cells. Accordingly, the transfected cells were washed twice with PBS and then were trypsinized for 3 min. The complete RPMI medium was added to the trypsinized cells, and then the cells were centrifuged for 5 min at 3000 rpm. The supernatant was discarded, and the pellet was resuspended in PBS for washing and incubated for 3 min in cold methanol for fixation. The cells were resuspended in PBS with Triton-X-100 (0.1%) and incubated for 3 min for permeabilization. For primary antibody staining, the cells were resuspended and incubated overnight at 4 °C in the PBS supplemented with 1% BSA and the diluted mouse monoclonal anti-NP (1-500) (Abcam, ab128193). After washing with pure PBS, the cells were centrifuged and resuspended in the PBS with 1% BSA and 1-1000 secondary antibodies (goat anti-mouse IgG, Alexa Fluor 594, Abcam, USA). The cells were then incubated in dark conditions for 2 h. The same instructions were followed for staining the cells with the primary and secondary antibodies against NS1 using rabbit monoclonal anti-NS1 (1-500) (Abcam, ab91642). and goat anti-rabbit IgG (1-1000) (Alexa Fluor 594, Abcam, USA) [32]. For staining MxA and STAT3, the primary antibodies; rabbit polyclonal anti-MxA (Novus Biologicals, NBP132905) and mouse monoclonal anti-STAT3 (Abcam, ab119352) were used. The same secondary antibodies; goat anti-rabbit (Alexa Fluor 488, Abcam) and goat anti-mouse (Alexa Fluor 594) has been used to achieve the kinetic expression of MxA and STAT3, respectively. Finally, the flow cytometry assay (BD Accuri 6 Plus) was used to assess the protein levels using a resuspended pellet in 500 µl PBS [33, 34].

### Western blot

The expression profile of viral NP and NS1 proteins, in addition to cellular MxA and STAT3 proteins was double-checked using an immunoblotting assay. Total protein was extracted from transfected and infected A549 cells using the complete lysis and extraction buffer, RIPA (ThermoFisher, USA). Then the protein was denaturized using a loading buffer containing 10% sodium dodecyl sulfate (SDS) and boiling at 95–100 °C for 5 min. 100 ng of denaturized protein was loaded in 10% sodium dodecyl sulfate-polyacrylamide gel, and the electrophoresis was carried out for 4 h at 75 V using the Bio-Rad Mini-Protean II electrophoresis unit. The protein bands were then transferred onto nitrocellulose membranes (Millipore, MA, USA) using Bio-Rad electro-blotting system (Bio-Rad Mini Trans-Blot Electrophoretic Transfer Cell). For blocking, the membrane was incubated for one hour at room temperature in 30 ml of Tris Buffered Saline containing 5% non-fat dry milk and 0.1% Tween-20 (pH 7.5). Then the membrane was individually incubated overnight at 4 °C with the previously described primary antibodies targeted NP, NS1, MxA and STAT3. The membrane was then washed twice using WesternBreeze solution 16x (Invitrogen, USA) followed by 2 h incubation at room temperature with mouse monoclonal anti- β-actin (Sigma, Hamburg, Germany). Finally, the membranes were washed twice and incubated for 2 h at RT with either anti-moue or anti-rabbit ready-to-use 2° Solution Alkaline-Phosphates (AP) Conjugated (Invitrogen, USA). After well washing, the chromogenic detection of expected bands was performed immediately using AP substrate (WesternBreeze, Invitrogen, USA).

### Enzyme-linked immunosorbent assay (ELISA)

ELISA assay was used to quantify the released IL-6 and IFN-β using human ELISA kits (Abcam 46,042 and Abcam 278,127, respectively). Accordingly, A549 cells were overnight cultured in a 96-well plate with a density of 10,000 cells/well. Then the cells were transfected using a 20 µl optimum medium that contains 25 µg/well of either miR-141 inhibitor or precursor suspended in 2 µl HyperFect. The transfected cells were incubated for 6 h; then, a fresh RPMI medium was added to each well instead of the transfection medium, and the cells were incubated for two days. Other cells were exposed to the IFN-β (5 IU/ml) for two hours before the IAV infection and continuously during the infection. Finally, the treated and transfected cells were infected with IAV, with MOI of 0.05, followed by an incubation period of (0, 6, 12, 24, and 48 h). At each time, 50 µl of the lysed cells were transferred into the ELISA plate and incubated for 3 h at room temperature with the same volume of the control solution and 1X biotinylated antibody. After washing, 100 µl of 1X streptavidin-HRP solution was added to each well which then was incubated for 30 min in the dark. Finally, 100 µl of the chromogen TMB substrate solution was added to each well, followed by 15 min incubation at RT away from the light. The stop solution was added, and the absorbance of each well was monitored using 450 nm [35].

### Data analysis

For quantification the cycle threshold (Ct) of each investigated gene expression, delta-delta-Ct equations were used as previously described: (1) delta-Ct = Ct value for gene - Ct value for GAPDH, (2) (delta–delta-Ct) = delta-Ct for experimental–delta-Ct for control, (3) relative expression of targeted gene = (2^− delta−delta ct^) [36, 37]. Statistical analysis was done using the student’s t-test between two groups. *P-*value ≤ 0.05 was considered statistically significant.

## Results

### IAV infection stimulates miR-141 to reduce MxA gene expression, potentially

**Fig. 1:**
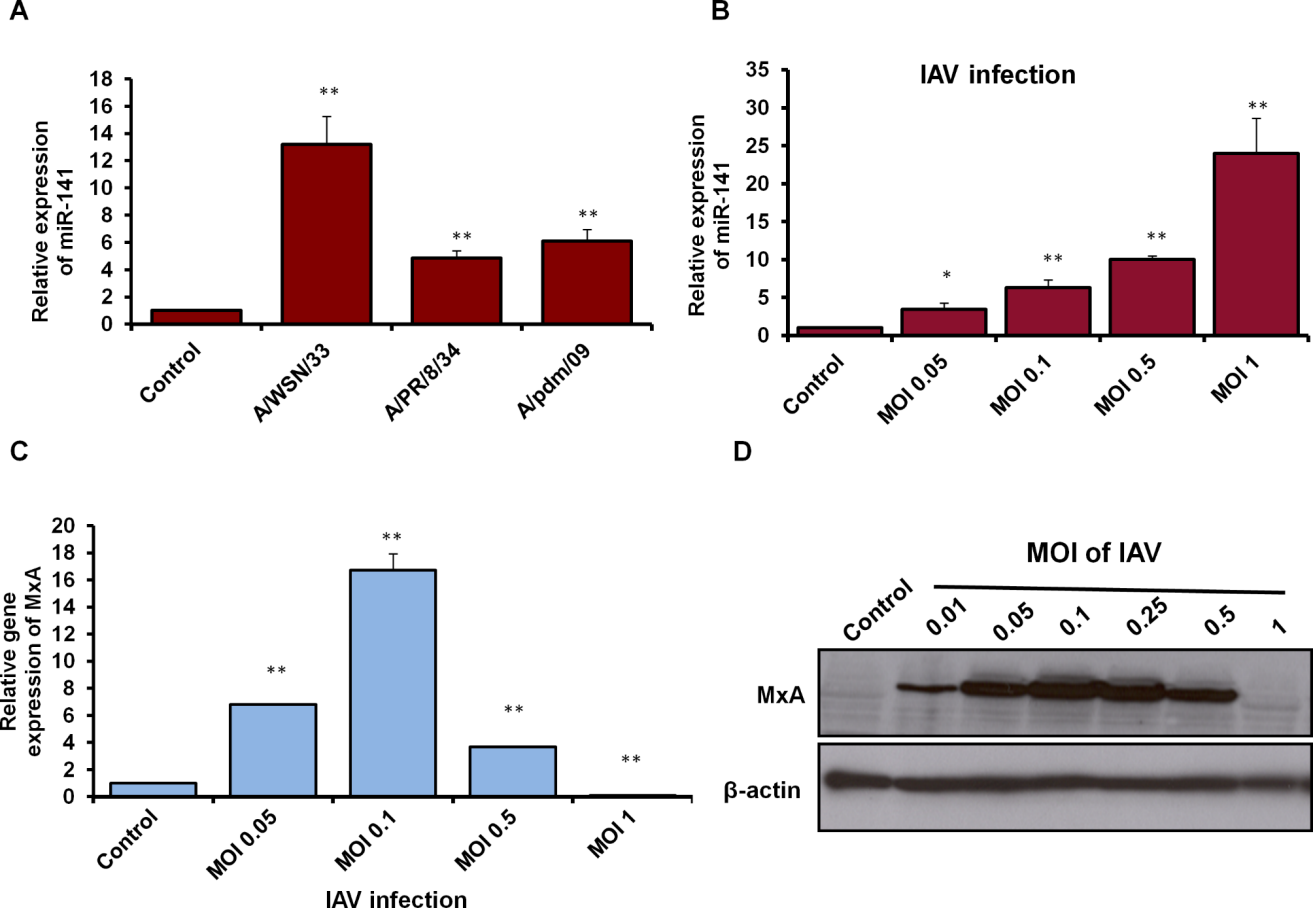
Expression profile of miR-141 and MxA in IAV-infected cells. **(A)** Relative expression level of miR-141 in A549 cells infected with different strains of IAV, including A/WSN/33, A/PR/8/34, and A/pdm/09 indicated by fold change using qRT-PCR. **(B)** Expression levels of miR-141 in A549 cells infected with different MOIs of IAV/WSN/33 indicated by qRT-PCR. Error bars indicate the standard deviation (STD) of three independent experiments. **(C)** Relative gene expression of MxA in A549 cells infected with IAV/WAN/33, different MOIs, for 24 h indicated by qRT-PCR. Error bars indicate the STD of three independent experiments. Student two-tailed *t*-test used for statistical analysis, (*) indicates *P* ≤ 0.05, while (**) indicates *P* ≤ 0.01. **(D)** Immunoblotting analysis of MxA protein expression in A549 cells infected with different MOIs of IAV/WSN/33 for 24 h, β-actin expression profile severed as an internal control

To investigate whether IAV infection induces the expression of miR-141 in infected cells, A549 cells were seeded in 6-well/plate and infected with different strains of IAV (MOI = 1), including A/WSN/33, A/PR/8/34, and A/pdm/09 followed by overnight incubation. Interestingly, the level of miR-141 significantly upregulated 12-fold changes upon infection with IAV/WSN/33 strain. In addition, the level of miR-141 increased up to 6-fold changes upon infection with influenza A/pdm/09 compared with non-infected cells (control), (Fig. 1A; Table 2). In addition, the expression level of miR-141 significantly upregulated in A549 cells infected with different MOIs of IAV/WSN/33, including MOI of 0.05, 0.1, 0.5, and MOI of 1 in a dose-dependent manner (Fig. 1B; Table 3). Furthermore, upon infection, we investigated the relative expression profile of MxA as an interferon-inducible gene and antiviral protein. Surprisingly, the relative MxA expression profile at both RNA and protein levels markedly increased upon the infection with low MOIs of IAV/WSN/33 indicated by qRT-PCR (Fig. 1C; Table 4). The immunoblotting analysis of MxA protein expression further confirmed the overexpression of MxA protein in A549 cells infected with low MOIs of IAV, while completely disappeared in cells infected with high MOIs, including MOI of 1, 1.5, and MOI of 2 (Fig. 1D and Supp. Figure 1). Together, these findings indicate thatthe infection with a high MOI of IAV strongly inhibits the expression profile of MxA, suggesting the ability of IAV to post transcriptionally regulate the expression of MxA may be via upregulated miR-141.

**Table 2:** Quantification analysis of miR-141 in A549 cells infected with IAV strains

Conditions	Expression fold changes	STD	*P values*
Control cells	1	00.00	
A/WSN/33	13.18**	2.09	0.014
A/PR/8/34	4.85**	0.56	0.010
A/pdm/09	6.1**	0.84	0.013

STD: Standard deviation

**: Indicates high significant *P* values ≤ 0.01

**Table 3:** Quantification analysis of miR-141 in A549 cells infected with IAV/WSN/33 strain

Conditions	Expression fold changes	STD	*P values*
Control	1	00.00	
MOI 0.05	3.4*	0.8	0.05
MOI 0.1	6.2**	1.04	0.01
MOI 0.5	9.9**	0.49	0.001
MOI 1	23.9**	4.6	0.01

STD: Standard deviation

*: Indicates significant *P* values ≤ 0.05

**: Indicates high significant *P* values ≤ 0.01

**Table 4:** Quantification analysis of MxA gene expression in infected A549 with IAV A/WSN/33 strain

Conditions	Expression fold changes	STD	*P values*
Control cells	1	00.00	
MOI 0.05	6.82**	0.01	0.0000
MOI 0.1	16.72**	1.23	0.0031
MOI 0.25	3.70**	0.07	0.0003
MOI 0.5	0.09**	0.02	0.0000

STD: Standard deviation

**: Indicates high significant *P* values ≤ 0.01

### Transfection of pre-miR-141 and specific inhibitor has no toxic effect in transfected cells independent of infection

**Fig. 2:**
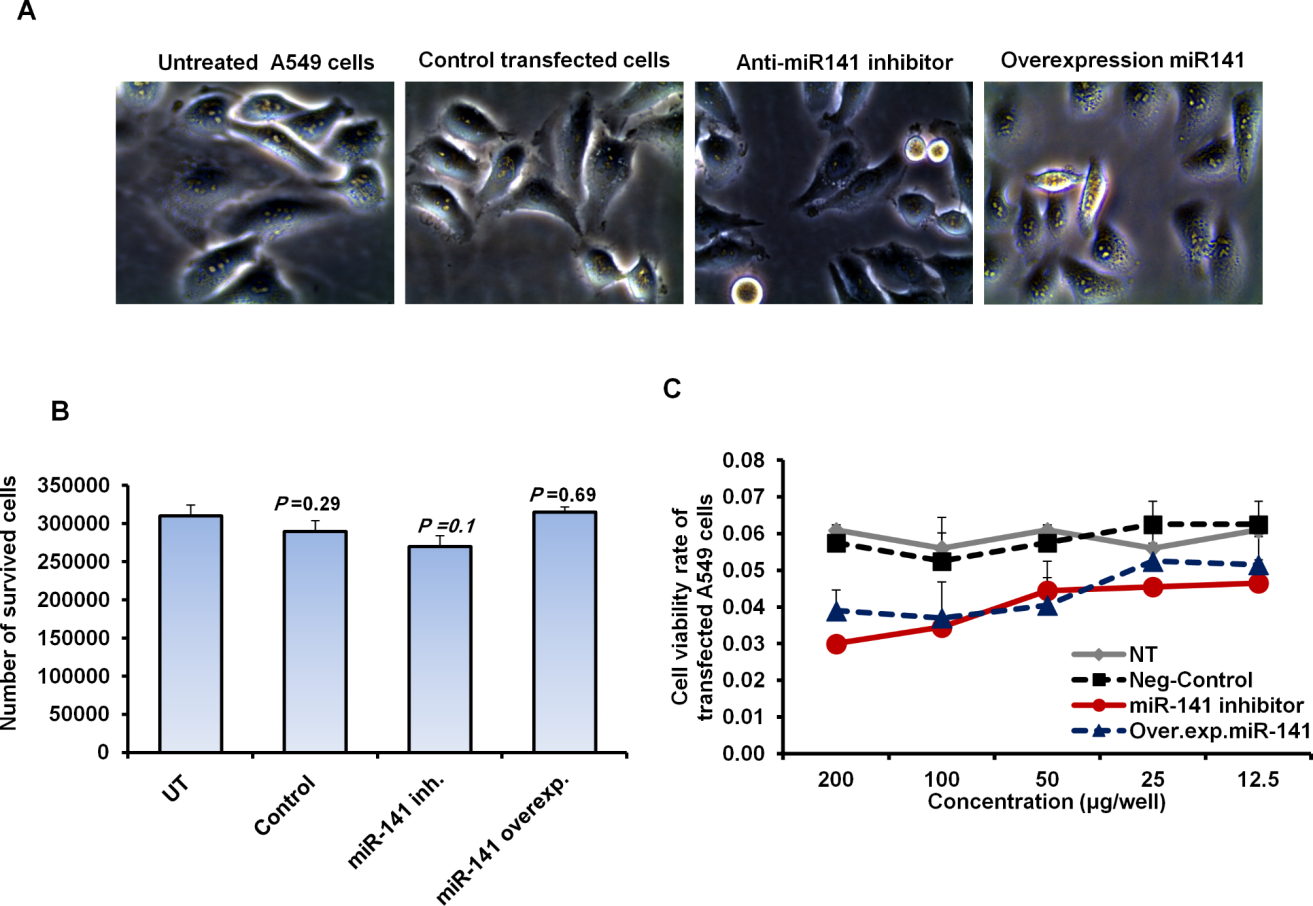
Cytotoxic influence of pre-miR-141 and specific inhibitor transfection in A549 cells. **(A)** A549 cell morphology indicated by inverted microscope upon 48 h of transfection with either pre-miR-141 or an inhibitor antagonist miR-141 compared with control-transfected and untreated cells. **(B)** After transfection with the miR-141 inhibitor or precursor, the number of living A549 cells statement. **(C)** Cell viability rate of transfected A549 cells with different concentrations of the pre-miR-141 and specific inhibitor indicated by the absorbance rate of treated cells with MTT agent. Error panels present the STD of three independent experiments. Student two-tailed *t*-test was used for statistical analysis

As shown in Fig. 2A, the transfected cells with pre-miR-141 or miR-141 inhibitor showed neglected differences in A549 cell proliferation rate when compared with control-transfected cells, evidenced by stable cell morphology. Furthermore, the number of living A549 cells transfected with either inhibitor antagonist miR-141 or pre-miR-141 was approximately impartial, with no significant differences compared with control-transfected cells and untreated cells (Fig. 2B). Likewise, cell viability rate of transfected A549 cells with miR-141 inhibitor or pre-miR-141 showed negligible differences in cell viability rate compared with control-transfected cells and untreated cells (Fig. 2C). These data demonstrate that cell viability, morphology and number of survived cells were maintained upon transfection, indicating that the transfection of the pre-miR-141 and miR-141 specific inhibitor has no cytotoxic effect in A549 cells independent of infection.

### MiR-141 effectively regulates IAV replication in transfected A549 cells

**Fig. 3:**
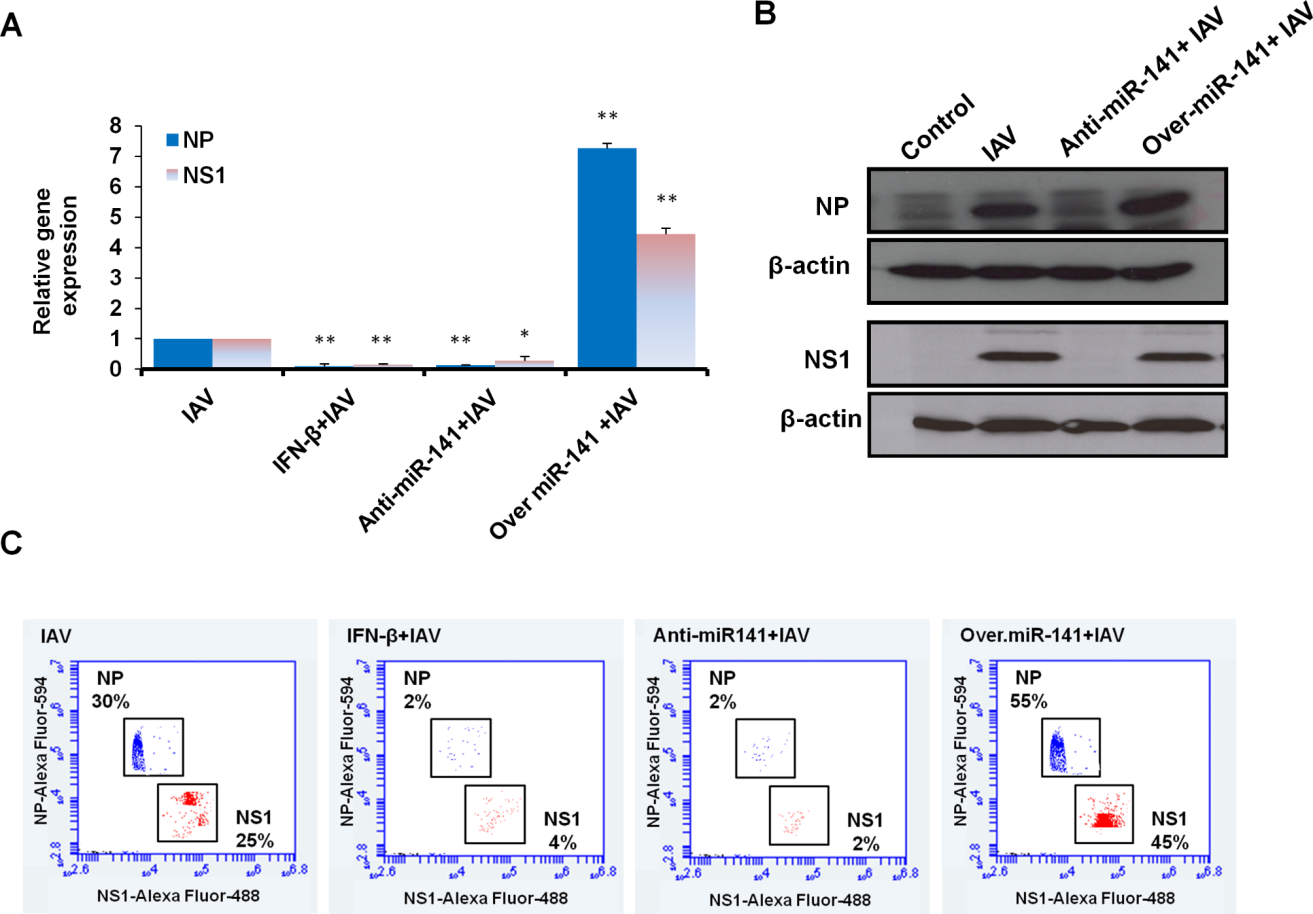
Expression profile of viral NP and NS1 in A549 cells transfected with pre-miR-141 or specific inhibitor. **(A)** Relative gene expression of both viral NP and NS1 in A549 cells treated with IFN-β or transfected with pre-miR-141 or specific inhibitor compared with noninfected cells (control cells). Error bars indicate the STD of three independent experiments. Student two-tailed *t*-test used for statistical analysis, (*) indicates *P-values* ≤ 0.05, and (**) indicates *P* ≤ 0.01. **(B)** Immunoblotting analysis of viral NP and NS1 protein expression in infected A549 that either treated with IFN-β or transfected with miR-141 overexpression vector or specific inhibitor compared with control cells, β-actin expression profile severed as an internal control. **(C)** Flow cytometric assay quantifies the kinetic proteins expression profile of NP (in blue dots) and NS1 (in red dots) in infected and transfected A549 cells compared with control cells

To investigate the molecular impact of miR-141 on IAV replication, miR-141 transfected cells were infected with influenza A/WSN/33 at a low multiplicity of infection (MOI 0.05) and overnight incubated. The expression profile of viral NP and NS1 of infected A549 cells was monitored using qRT-PCR. Consequently, the relative expression of NP and NS1 was dramatically increased, up to 7 and 5 folds respectively, in miR-141 overexpressing cells compared with what was seen in cells transfected with miR-141 inhibitor and control cells. Notably, the cells pretreated with IFN-β showed, as expected, strong reduction in viral NP and NS1 expression as elicited in A549 cells that transfected with an inhibitor antagonist miR-141 (Fig. 3A; Table 5). Interestingly, the protein expression profile of both viral NP and NS1 was strongly depleted in A549 cells transfected with the inhibitor antagonist miR-141, while their expression markedly increased in cells transfected with pre-miR-141 indicated by immunoblotting (Fig. 3B and Supp. Figure 2). Likewise, the kinetic protein expression of viral NP and NS1 markedly reduced in cells transfected with the miR-141 inhibitor as well as in cells treated with IFN-β, indicated by flow cytometry, since their expression has been detected in only 0.2% and 4% of stained cells (Fig. 3C). However, the protein expression profile of both NP and NS1 showed an evident expression in more than 55% and 45% of stained cells transfected with the pre-miR-141 compared with control-transfected cells, as presented in Fig. 3C. This observation suggested that both the inhibitor antagonist miR-141 and IFN-β can likely inhibit IAV replication. Together, these data corroborate that miR-141 plays a role in maintaining the replication cycle of IAV.

**Table 5:** Steady state of viral NP and NS1 gene expression transfected A549 cells

Genes	Condition	Expression fold changes	STD	*P*-values
NP	IAV	1.00	0.00	
	IFN-β + IAV	0.10**	0.09	0.004
	Anti-miR-141 + IAV	0.13**	0.02	0.002
	Over. miR-141 + IAV	7.27**	0.16	0.003
NS1	IAV	1.00	0.00	
	IFN-β + IAV	0.14**	0.03	0.008
	Anti-miR-141 + IAV	0.27*	0.16	0.02
	Over. miR-141 + IAV	4.44**	0.21	0.001

**NT** nontreated cells

**STD** Standard deviation of three independent experiments

*: Indicates significant *P* values ≤ 0.05

**: Indicates high significant *P* values ≤ 0.01

### Microarray analysis represents miR-141-regulated genes, including *MxA, STAT3, LAMP3* and *IFI 27* gene family in A549 cells

**Fig. 4:**
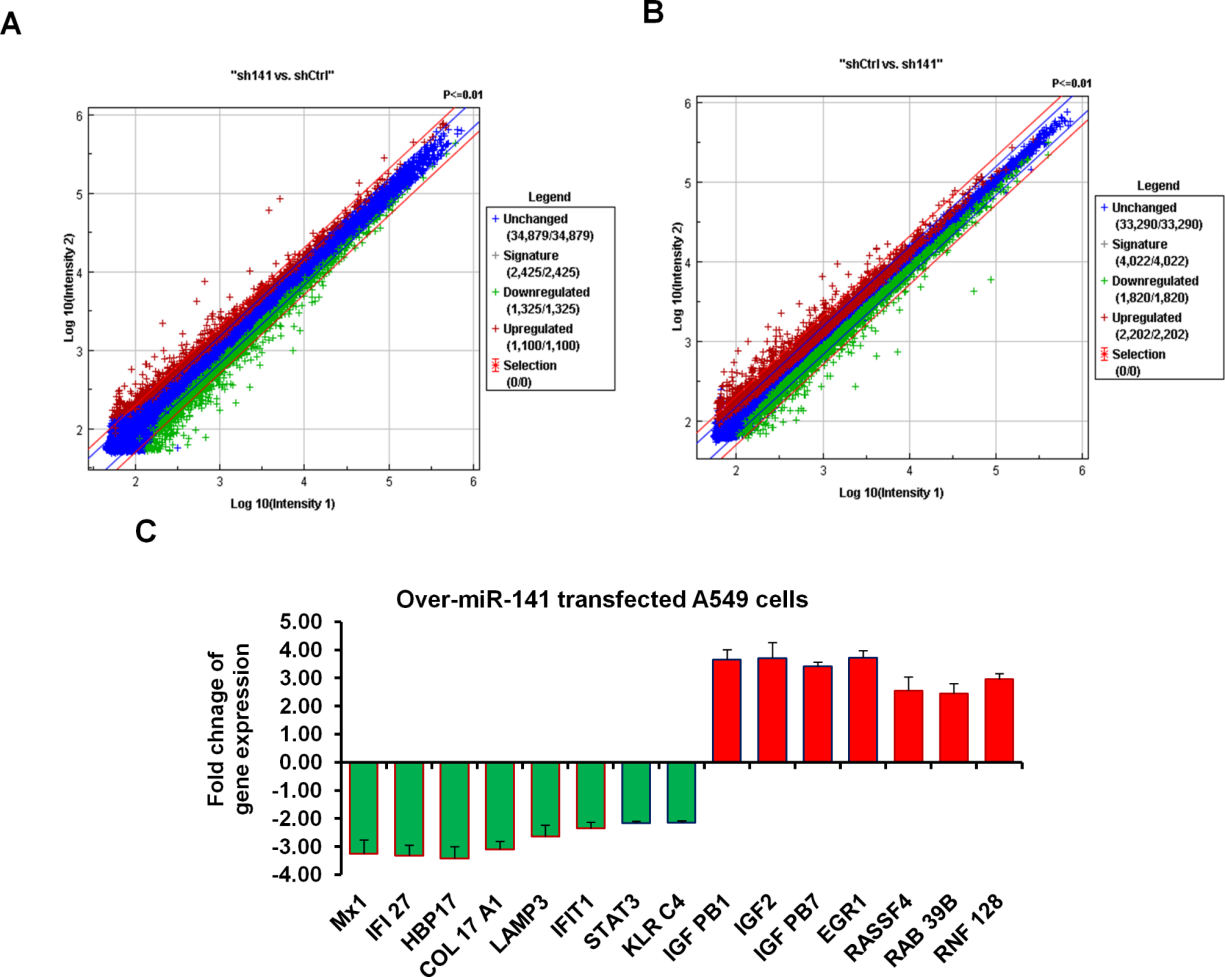
Microarray analysis of transfected A549 cells with pre-miR-141. **(A)** Microarray analysis of gene expression in A549 cells transfected with pre-miR-141 overexpression vs. control-transfected cells using a miRNA library from Exiqon. **(B)** Microarray analysis of gene expression in A549 cells transfected with pre-miR-141 overexpression vs. control-transfected cells using oligonucleotides from Ambion. The blue color indicates the sustained gene expression, the red indicates the upregulated genes, and the green indicates the downregulated genes. **(C)** The expression of the most relevant genes to an antiviral strategy indicates the upregulated genes in red columns and downregulated genes in green. Error bars indicate the STD between Exiqon and Ambion data

As presented in Fig. 4 (A and B), the microarray data of transfected A549 cells with pre-miR-141 showed that dozens of mRNAs whose expression were negatively affected by the presence of high levels of miR-141 indicated by both Exiqon and Ambion chips. However, many mRNAs were not affected by the level of miR-141 in A549 cells. On the contrary, the expression levels of many genes were downregulated in response to the high level of miR-141. Notably, downregulated genes by miR-141 enriched many transcripts mRNA subject to virus repression, including the *MX1* encoding for myxovirus resistance protein, *IFI27* encoding for interferon alpha inducible protein 27, *LAMP3* encoding for lysosomal associated membrane protein3, signal transducer and activator 3 (*STAT3*) and killer like receptor C4 (*KLRC4)* (Fig. 4C). Alternatively, the most increased mRNAs were linked with virus replication and cell development, such as *IGFBP*-*1* encoding for insulin-like growth factor binding protein 1, *IGF2* encoding for Insulin-like growth factor II, (*IGFBP-7*), *EGR1* encoding for early growth response protein1 and *RASSF4* encoding the RAS-association domain family (Fig. 4C). These data indicate the molecular impact of miR-141 to ensure IAV replication and suggest the possible regulatory role of miR-141, if increased, in the regulation of *Mx1*, *IFI27*, *HBP17*, *LAMP3*, *STAT3*, and *KLRC410* gene expression in A549 cells.

### MiR-141 modulates the expression of *MxA* and *STAT3* in transfected A549 cells

**Fig. 5:**
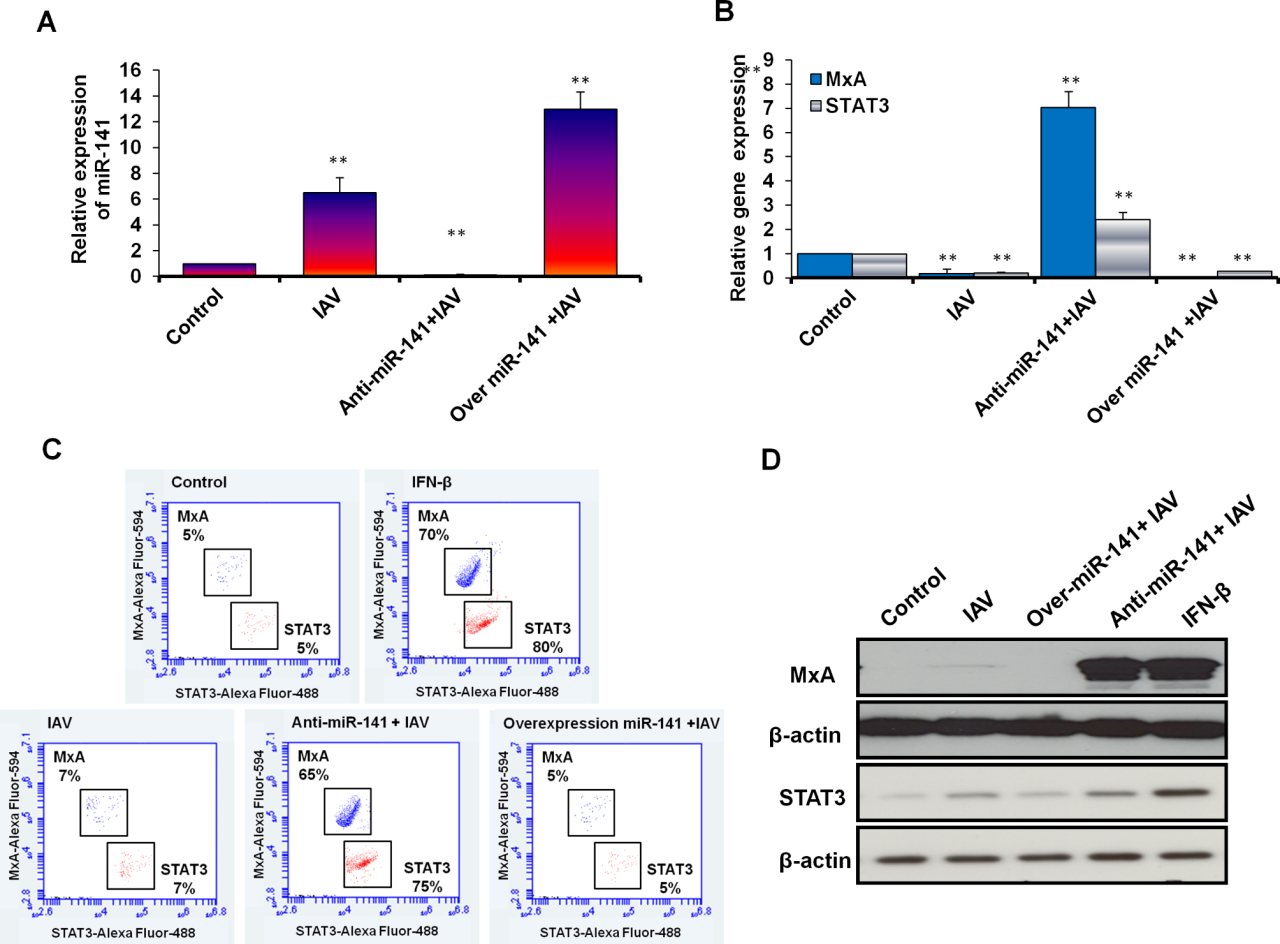
The correlation between miR-141 level and the expression profile of MxA and STAT3 in infected A549 cells. **(A)** Quantification of steady-state miR-141 in infected A549 cells with MOI of 0.5 and transfected with either pre-miR-141 or miR-141 inhibitor compared with noninfected cells (control) using qRT-PCR. **(B)** Relative gene expression of MxA and STAT3 in infected A549 cells transfected with either specific inhibitor against miR-141 or pre- miR-141 compared with control-transfected cells using qRT-PCR. Error bars indicate the STD of three independent experiments. Student two-tailed *t*-test used for statistical analysis, (*) indicates *P-values* ≤ 0.05, and (**) indicates *P* ≤ 0.01. **(C)** Flow cytometric assay quantifies the kinetic proteins expression profile of MxA (in blue dots) and STAT3 (in red dots) in infected and transfected A549 cells compared with control cells. **(D)** Western blot analysis reveals the protein expression level of MxA and STAT3 in infected and transfected cells compared to control cells, β-actin expression profile severed as an internal control

We confirmed the knockdown efficiency and alteration of miR141 expression level in transfected A549 cells indicated by the fold change using qRT-PCR. The relative expression of miR-141 significantly upregulated 12 folds in cells transfected with the pre-miR-141. In contrast, its expression level dramatically dropped in cells transfected with the miR-141 inhibitor compared to control-transfected cells (Fig. 5A; Table 6). To check the possible regulation of *MxA* and *STAT3* expression by miR-141, the expression profile of *MxA* and *STAT3* was quantified in infected and transfected A549 cells with the inhibitor antagonist miR-141 and pre-miR-141 using qRT-PCR, flow cytometry, and immunoblotting assay. Surprisingly, the relative gene expression of both *MxA* and *STAT3* strongly increased 7 folds and 3 folds respectively in cells transfected with miR-141 inhibitor, while their expression dramatically decreased in miR-141 transduced cells (Fig. 5B; Table 6). Furthermore, the kinetic protein expression of MxA and STAT3 markedly depleted in miR-141 transduced cells, indicated by flow cytometry, since their expression was detected within only 5% of stained cells (Fig. 5C). However, the protein expression profile of both MxA and STAT3 markedly resorted in more than 65% of stained cells transfected with the inhibitor antagonist miR-141, like their expression profile in cells pretreated with IFN-β, as presented in Fig. 5C. The double-check of MxA and SATA3 protein expression profiles by western blot further confirmed the decreasing of both proteins in infected A549 cells transfected with pre-miR-141 compared with control-transfected and nontreated cells. In contrast, their expression showed full recovery in cells transfected with the inhibitor antagonist miR-141 (Fig. 5D and Supp. Figure 3). These results indicate the potential regulation of both MxA and STAT3 by miR-141 and the ability of miR-141 inhibitor to restore their expression profile in infected A549 cells, which subsequently disturbs IAV replication.

**Table 6:** Quantification analysis of miR-141, MxA, and STAT3 in transfected and infected A549 cells

Genes		Condition	Expression fold changes	STD	*P*-values
miR-141		Control	1.00	0.00	
		IAV	6.52*	1.16	0.021
		Anti-miR-141 + IAV	0.15**	0.03	0.007
		Over.miR-141 + IAV	12.97**	1.35	0.006
MxA STAT3		Control	1.00	0.00	
		IAV	0.19*	0.18	0.02
		Anti-miR-141 + IAV	7.04**	0.65	0.005
		Over.miR-141 + IAV	0.02**	0.01	0.001
		Control	1.00	0.00	
		IAV	0.20**	0.05	0.001
		Anti-miR-141 + IAV	2.41*	0.29	0.020
		Over.miR-141 + IAV	0.28**	0.01	0.001

**STD** Standard deviation of three independent experiments

*: Indicates significant *P* values ≤ 0.05

**: Indicates high significant *P* values ≤ 0.01

### Deception of miR-141 successfully modulates the production of IFN-β and IL-6 in transfected A549 cells

**Fig. 6:**
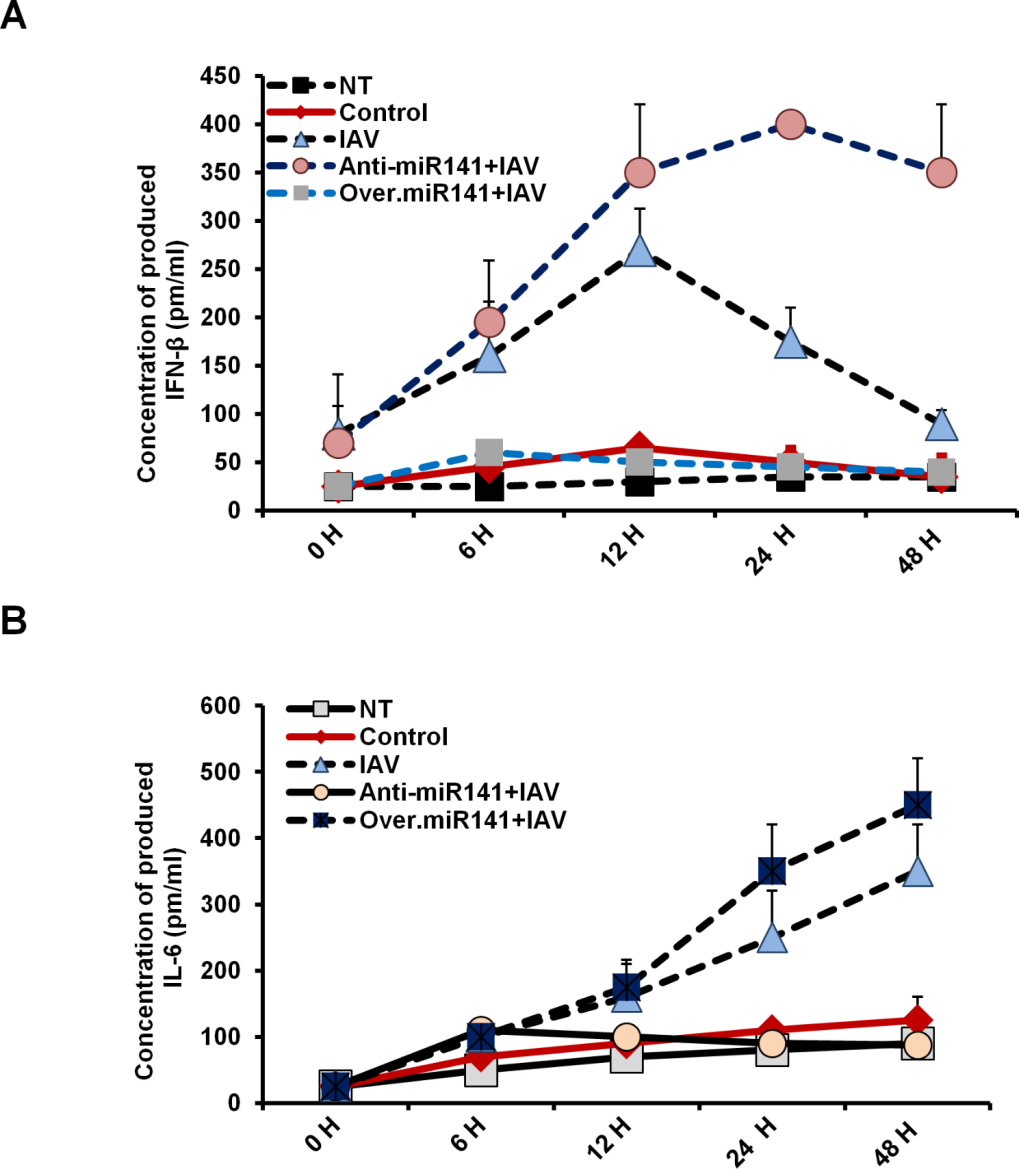
Levels of produced cytokines in infected and transfected A549 cells. **(A)** The concentration of produced IFN-β (pm/ml) in the fluid media of infected and transfected A549 cells at the indicated time points in comparison with its concentration in the fluid media of nontreated cells (NT) and cells treated with the transfection reagent (Control). **(B)** The concentration of IL-6 in the culture media of infected and transfected A549 cells at the indicated time points compared with its concentration in the fluid media of NT cells and control-treated cells. Error bars indicate the STD of four different replicates

To investigate the relationship between miR-141 expression level and cytokine production upon infection, the concentration of secreted IFN-β and IL-6 was monitored in transfected A549 cells in a time-course experiment. As shown in Fig. 6A, the concentration of produced IFN-β markedly increased in infected A549 cells transfected with miR-141 inhibitor up to350 pm/ml at 24 h post-infection. In contrast, the level of produced IFN-β significantly decreased in cells transfected with pre-miR-141 compared with control transfected cells. This observation indicates the molecular impact of miR-141 in regulating IFN signaling during IAV infection. Unlike IFN-β, the level of produced IL-6 markedly increased up to 400 pm/ml in miR-141 transduced cells at 24 h post-infection, while significantly decreased in cells transfected with the inhibitor antagonist miR-141, indicating the role of miR-141 in IL-6 signaling pathway in infected cells (Fig. 6B). Taken together, these data strongly suggest the involvement of miR-141 in the mechanism by which IAV regulates IFN-β signaling pathway and cellular immune response following infection.

## Discussion

As a member of the miR-200 family, miR-141 is typically attributed to the host immune signaling, inflammatory functions, cell differentiation, and cell cycle, contributing to many cellular processes such as cell proliferation, apoptosis, and antiviral defenses [38]. Accordingly, some miRNAs are induced to regulate viral pathogenesis via modulating pro-inflammatory signaling pathways during infection. For instance, miR-29c and miR-451 are recovered upon IAV infection to suppress the inflammatory responses of cells [39, 40]. As indicated in a pilot study, miR-141 was significantly expressed upon infection with influenza virus H5N1 to downregulate transforming growth factor beta 2 (TFG-β2) in infected cells [41]. Likeminded, the present study powerfully reveals that infection with influenza virus H1N1 stimulated the expression of cellular miR-141. Once overexpressed in A549 cells, miR-141 inhibits the translation of MxA and STAT3 following IAV infection and IFN-β treatment. Deception of miR-141 in infected cells significantly inhibits viral replication via restoring MxA and STAT3 expression profiles. Importantly, our findings exhibit the possible involvement of miR-141 in IAV-mediated interferon signaling upon infection to ensure viral replication. Furthermore, the microarray data provided here clearly shows the ability of miR-141 to regulate a variety of antiviral gene expressions in transfected A549 cells. Among the targeted mRNAs affected almost three-fold change in miR-141 transduced cells were interferon alpha inducible protein 27 *(IFI27)* and lysosomal associated membrane protein3 (*LAMP3)* mRNAs.

As a cellular immune response to IAV infection, IL-6 is a pleiotropic cytokines produced in response to cell injury and infection [42]. Once it targets its specific receptor, IL-6 stimulates a signaling cascade associated with JAK/STAT3 activation pathway. JAK/STAT signaling promotes the transcription of multiple downstream genes related to cellular signaling processes, including cytokines, receptors, adaptor proteins, and protein kinases, including GADD45 beta, SOCS1, MAP3K8, SOCS3, and Mx1 [43]. The number of genes regulated by IL-6 activity may explain the pleiotropic nature of this interleukin [44]. Accordingly, the biological consequences of IL-6 production have been associated with both pro- and anti-inflammatory effects, highlighting IL-6’s pivotal role in the activation and regulation of the immune response [45]. Based on this, we hypothesized that targeting STAT3 by upregulated miR-141 disturbs the function of produced IL-6 during IAV infection by inhibiting its related gene expression. In addition, the increasing level of produced IL-6 in infected cells pre-transfected with miR-141 mimic is due to the exponential replication of IAV, unlike the cells pre-transfected with an inhibitor antagonist miR-141.

On the other hand, IAV deceives host cellular processes to replicate successfully. Thus, the influenza virus inhibits the synthesis of host proteins, facilitates the expression of its proteins, and often correlates pathogenicity with growth rate. It is well known that IAV enters the host by binding to viral HA and sialic acid as an initial receptor [22, 46]. Once the virion enters the cells, RNA helicase retinoic acid-inducible gene I (RIG-I) expression is induced, and a critical signaling cascade is stimulated, followed by transcription of IFN-β [17]. IFN-β activates the STATs complex leading to the initiation of a defined cell signaling, the regular JAK/STAT signaling pathway [47]. Secretion of IFN-β and/or IFN-α from infected cells initiates an event that stimulates the expression of IFN-dependent genes [18]. In this pathway, JAKs associate with the IFN-β receptor, and subsequently, STATs are phosphorylated on conserved tyrosine residues and released from the receptor, where conformational changes lead to homo- (type 1, II, and III IFN). This change exposes a nuclear localization signal that facilitates nuclear translocation. Once in the nucleus, STATs binds to a specific nucleotide sequence known as interferon-stimulated regulatory element (ISRE) and serve as transcriptional activators that drive ISG expression, including *PKR, IGS15*, and *MxA* [48, 49]. The human myxovirus resistance protein A (MxA) is the central antiviral molecule that protects cells from infection and apoptosis. The human MxA protein encoded by the interferon-inducible *MX1* gene is an intracellular anti-IAV factor. The site of *MX1* is located on the long arm of chromosome 21 and contains 17 exons extending over 33 kb [50]. MxA is considered a large GTPase, which mediates broad resistance to influenza and other viruses in cell culture. Overexpression of the MxA protein has been found to perturb the trafficking and transferring of viral protein resulting in its accumulation in cells [49].

Mechanistically, several studies showed that the IAV-NS1 protein is a viral regulator of cellular gene expression via inhibiting the pre-mRNA process [51, 52]. In addition, viral NS1, which has RNA-binding activity, inhibits RIG-I’s activation and prevents interferon signaling pathways in infected cells[53]. Similarly, we confirmed that interferon production is stimulated following IAV infection as a cellular immune response; however, IAV can prevent further interferon production lately. Accordingly, the expression of several interferon-inducible genes is successfully induced in infected cells, including *MxA* and *STAT3*, which are targeted by upregulated miR-141. These findings suggest the role of miR-141 in blocking the JAK/STAT singling pathway following infection. Based on this, we hypothesize that IAV infection stimulates the expression of miR-141 in infected cells to regulate the expression profile of *MxA* as an interferon-inducible gene and *STAT3*, which is stimulated upon infection in response to IL-6 production.

## Conclusion

Influenza viruses have evolved strategies to counteract their hosts’ innate and adaptive defense responses. Induction of interferon (IFN)-stimulated genes plays a crucial role in protection against viral attack. Infection of influenza viruses usually results in up-regulating the potent IFN-regulated antiviral host factors, like the myxovirus resistance (Mx) proteins and other IFN-inducible genes. As a countermeasure, influenza viruses actively suppress the human ortholog of the Mx protein (MxA); however, the mechanism underlying this suppression remains poorly understood. Host microRNAs (miRNAs), around 22 nucleotides in length, act as global regulators of gene expression in mammalian cells and thus constitute attractive targets for viruses to usurp host cell functions. The current study deeply investigated the possible contribution of cellular miR-141 to IAV replication. The microarray data in A549 cells overexpressed miR-141 indicated the downregulation of different antiviral genes, including *MxA* and *STAT3*. Interestingly, the overexpression of miR-141 in infected A549 cells can increase the ability of IAV to ensure its replication cycle. Conversely, inhibition of miRNA-141 significantly reduced viral replication. Notably, we evidenced that the antiviral genes *MxA* and *STAT3* were subverted in a miRNA-141-dependent manner during the IAV infection of A549 cells. In contrast, there are induced in adjacent, noninfected cells due to interferon production from infected cells. Deception of miR-141 in infected cells restored the expression profile of both *MxA* and *STAT3*, leading to a marked disturbance of IAV replication. Notably, the level of miR-141 in infected A549 cells incorporated in the produced IFN-β and IL-6. Together, these data uncover an efficient miRNA-based mechanism that protects the influenza virus from a crucial antiviral host response and identifies miR-141 as a credible therapeutic target.

### Electronic supplementary material

Below is the link to the electronic supplementary material.

Supplementary Material 1

## Data Availability

All data supporting these findings are available at any time upon request to the corresponding author.

## List of Abbreviations

IAV: Influenza A virus
IFN-β: Interferon-beta
IFI27: Interferon alpha inducible protein 27
IL-6: Interleukin 6
ISGs: IFN-stimulated genes
LAMP3: Lysosomal associated membrane protein3
RIG-I: Retinoic acid-inducible gene I
RPMI: Roswell Park Memorial Institute
TFG-β2: Transforming growth factor beta 2
MDCK: Madin-Darby canine kidney
MOI: Multiplicity of infection
