# Supplementary material for: Influenza a virus regulates interferon signaling and its associated genes; MxA and STAT3 by cellular miR-141 to ensure viral replication
Source: Virol J. 2023 Aug 18;20:183. doi: 10.1186/s12985-023-02146-4 (PMC10439583; doi:10.1186/s12985-023-02146-4)
Supplement: Supplementary file 1 — Supplementary Material 1 [file 12985_2023_2146_MOESM1_ESM.docx]

**Supplementary data**


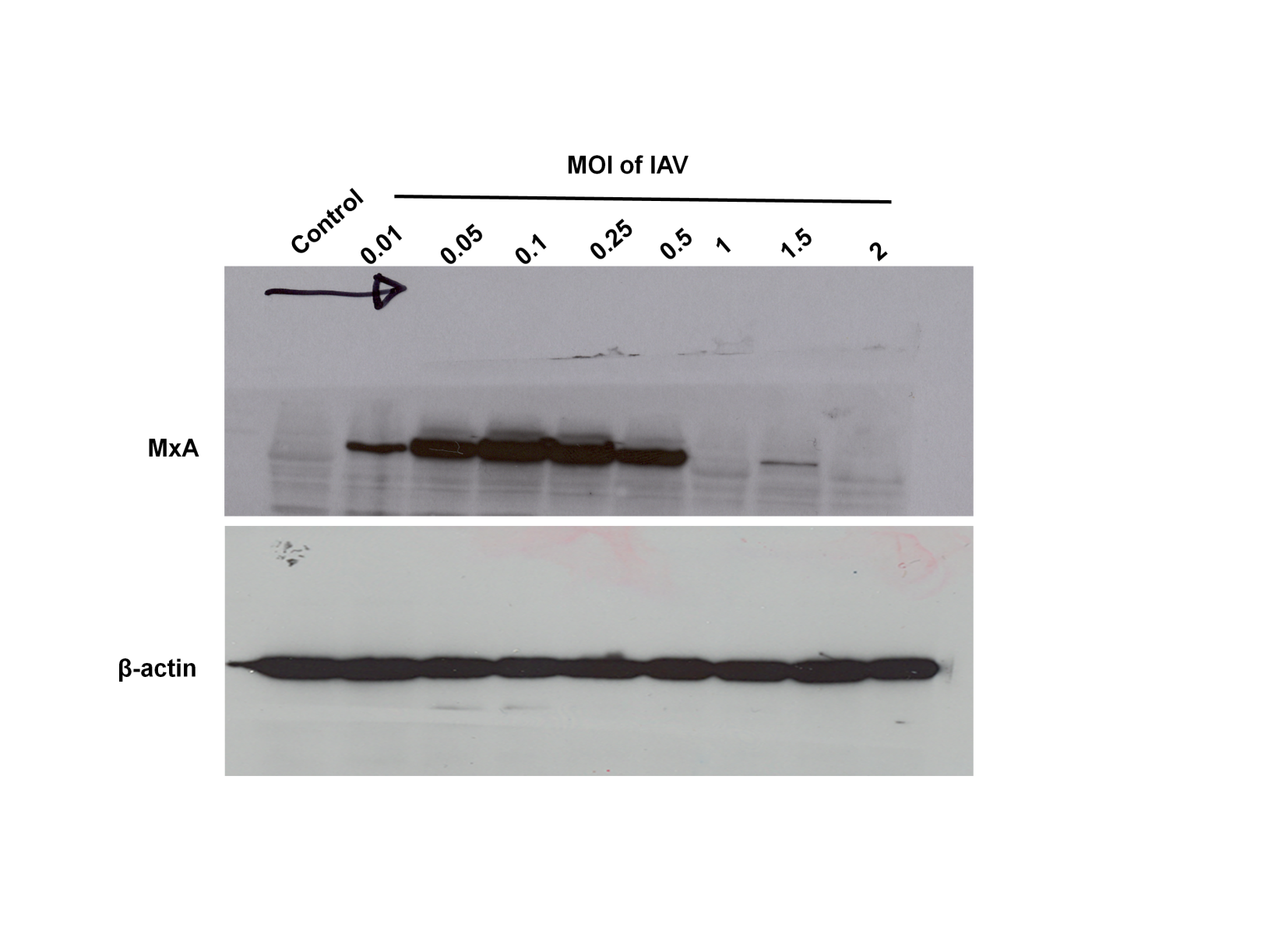


**Supp. Figure 1:**  Original data of immunoblotting reveals the protein expression levels of MxA and β-actin in A549 cells infected with different concentration of IAV/WSN/. β-actin was introduced as an internal control.


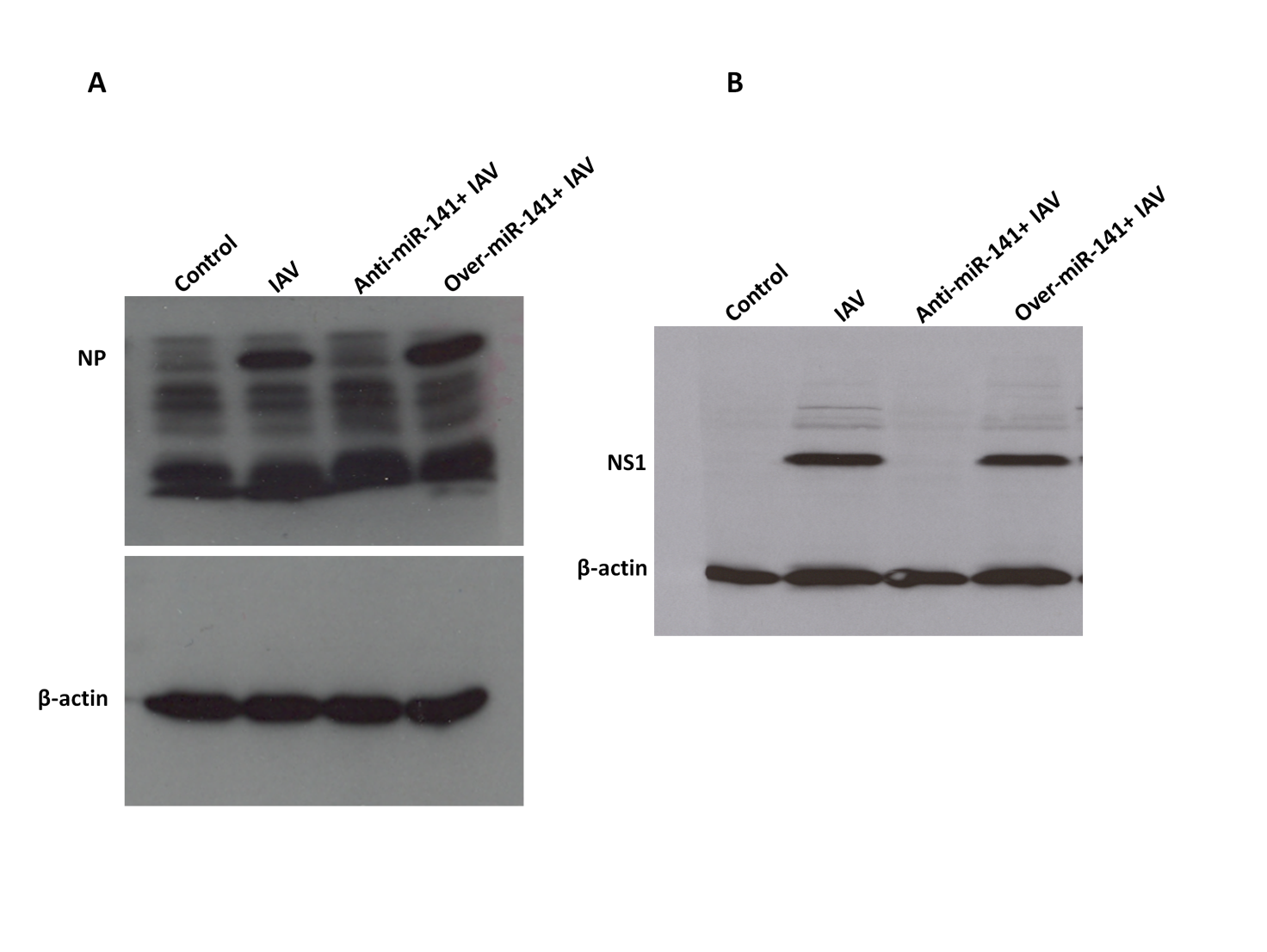


**Supp. Figure 2:** Original data of the immunoblotting reveals the protein expression profile of viral NP (A) and viral NS1 (B) A549 cells that transfected with an inhibitor antagonist miR141 or the pre-miR-141 compared to nontransfected. β-actin was introduced as an internal control.


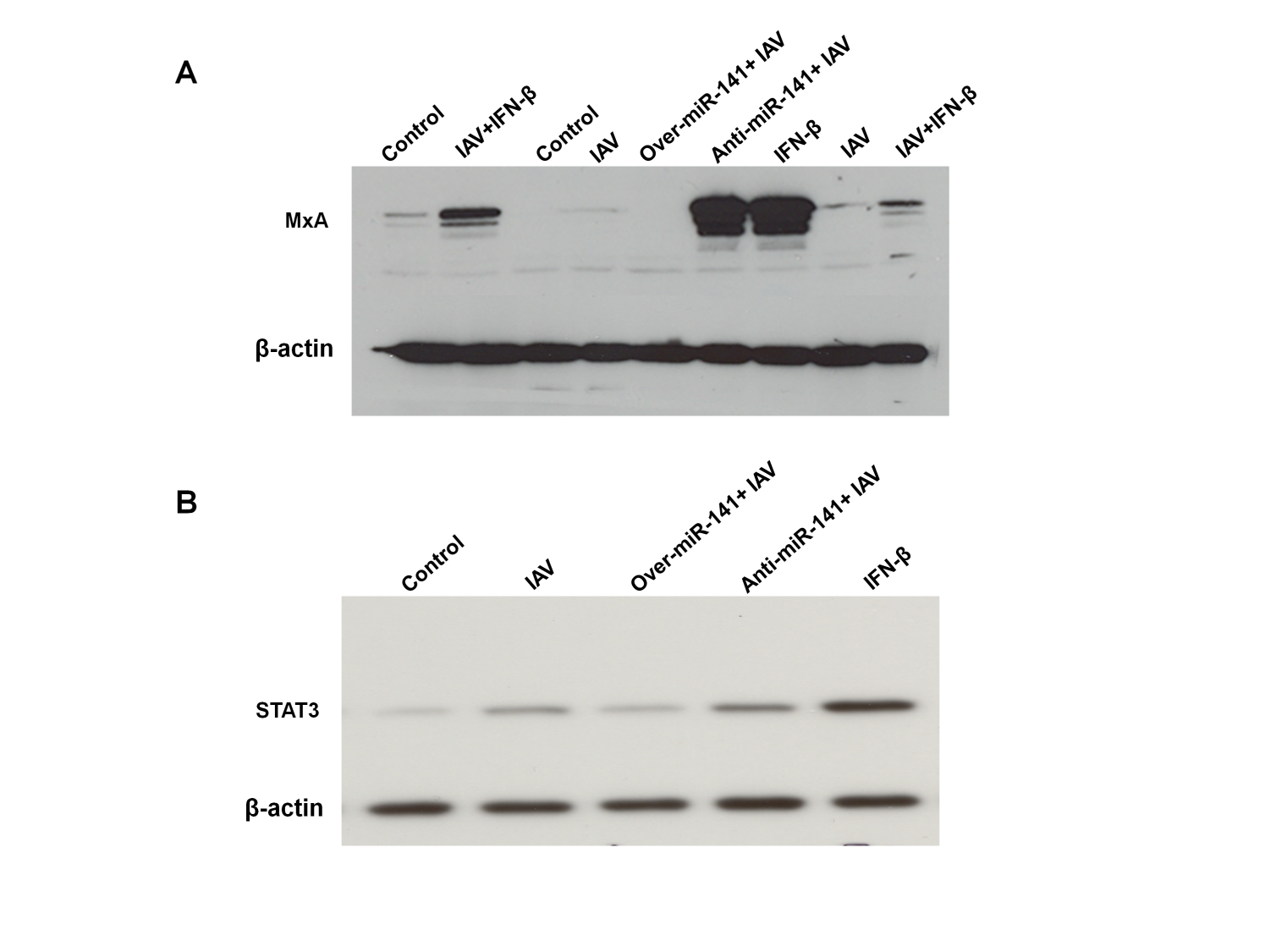


**Supp. Figure 3:** Original data of Ceacam 3 (CAM3) immunoblotting reveals the protein levels of CAM3 and β-actin in HeLa cells that transfected with an inhibitor antagonist miR141 or the miR-141 overexpression vector compared to nontransfected cells and other control transfected cells. β-actin was introduced as an internal control. β-actin was introduced as an internal control.
